# Trends in Postoperative Intensity-Modulated Radiation Therapy Use and Its Association With Survival Among Patients With Incompletely Resected Non–Small Cell Lung Cancer

**DOI:** 10.1001/jamanetworkopen.2022.30704

**Published:** 2022-09-08

**Authors:** Brian Yu, Sung Jun Ma, Olivia Waldman, Cynthia Dunne-Jaffe, Udit Chatterjee, Lauren Turecki, Jasmin Gill, Keerti Yendamuri, Austin Iovoli, Mark Farrugia, Anurag K. Singh

**Affiliations:** 1 Jacobs School of Medicine and Biomedical Sciences, University at Buffalo, The State University of New York, Buffalo; 2 Department of Radiation Medicine, Roswell Park Comprehensive Cancer Center, Buffalo, New York; 3 University at Buffalo, The State University of New York, Buffalo

## Abstract

**Importance:**

National guidelines allow consideration of postoperative radiation therapy (PORT) among patients with incompletely resected non–small cell lung cancer (NSCLC). However, there is a paucity of prospective data because recently completed trials excluded patients with positive surgical margins. In addition, unlike for locally advanced NSCLC, the role of intensity-modulated radiation therapy (IMRT) for PORT remains unclear.

**Objective:**

To evaluate trends of IMRT use for PORT in the US and the association of IMRT with survival outcomes among patients with incompletely resected NSCLC.

**Design, Setting, and Participants:**

This retrospective cohort study used data from the National Cancer Database for patients diagnosed between January 2004 and December 2019 with incompletely resected NSCLC who underwent upfront surgery with positive surgical margins followed by PORT.

**Exposures:**

IMRT vs 3D conformal radiation therapy (3DCRT) for PORT.

**Main Outcomes and Measures:**

The main outcome was overall survival. Multivariable Cox proportional hazards regression assessed the association of IMRT vs 3DCRT with overall survival. Multivariable logistic regression identified variables associated with IMRT. Propensity score matching (1:1) was performed based on variables of interest.

**Results:**

A total of 4483 patients (2439 men [54.4%]; median age, 67 years [IQR, 60-73 years]) were included in the analysis. Of those, 2116 (47.2%) underwent 3DCRT and 2367 (52.8%) underwent IMRT. Median follow-up was 48.5 months (IQR, 31.1-77.2 months). The proportion of patients who underwent IMRT increased from 14.3% (13 of 91 patients) in 2004 to 70.7% (33 of 471 patients) in 2019 (*P* < .001). IMRT was associated with improved overall survival compared with 3DCRT (adjusted hazard ratio, 0.84; 95% CI, 0.78-0.91; *P* < .001). Similar findings were observed for 1463 propensity score–matched pairs; IMRT was associated with improved 5-year overall survival compared with 3DCRT (37.3% vs 32.2%; hazard ratio, 0.88; 95% CI, 0.80-0.96; *P* = .003). IMRT use was associated with receipt of treatment at an academic facility (adjusted odds ratio [aOR], 1.15; 95% CI, 1.00-1.33; *P* = .049), having T4 stage tumors (aOR, 1.50; 95% CI, 1.13-1.99; *P* = .005) or N2 or N3 stage tumors (aOR, 1.25; 95% CI, 1.04-1.51; *P* = .02), and receipt of pneumonectomy (aOR, 1.35; 95% CI, 1.02-1.80; *P* = .04).

**Conclusion and Relevance:**

This cohort study found that use of IMRT for PORT among patients with incompletely resected NSCLC increased in the US from 2004 to 2019 and was associated with improved survival compared with 3DCRT. Further studies are warranted to investigate the role of different radiation therapy techniques for PORT.

## Key Points

**Question:**

What is the trend of intensity-modulated radiation therapy (IMRT) use for postoperative radiation therapy in the US, and what is its association with survival among patients with incompletely resected non–small cell lung cancer (NSCLC)?

**Findings:**

In this cohort study involving 4483 US patients with NSCLC, IMRT use increased from 2004 to 2019 and was associated with improved survival outcomes compared with 3D conformal radiation therapy.

**Meaning:**

The findings suggest that additional studies are warranted to investigate the role of IMRT for postoperative radiation therapy.

## Teaser

This cohort study assesses trends in use of postoperative intensity-modulated radiation therapy (IMRT) in the US and the association of IMRT with survival among patients with incompletely resected non–small cell lung cancer (NSCLC).

## Introduction

Positive margins after resection for non–small cell lung cancer (NSCLC) are associated with a poor prognosis and worse survival outcomes.^1^ This problem persists despite substantial advances in systemic therapy agents. For instance, the CheckMate 816 trial of neoadjuvant nivolumab and chemotherapy for resectable NSCLC reported a 14% positive margin rate.^2^ The National Comprehensive Cancer Network guideline allows consideration of postoperative radiation therapy (PORT) among patients with incompletely resected NSCLC.^3^ The recently reported LungART and PORT-C trials excluded patients with positive margins.^4,5^

Prior analysis of the National Cancer Database (NCDB) showed an overall survival (OS) benefit associated with PORT but did not compare the impact of radiation techniques.^6^ The role of IMRT for PORT remains unclear. To address this knowledge gap, we performed a cohort study using a national clinical oncology database to assess the trend of IMRT use in the US and its association with OS compared with 3D conformal radiation therapy (3DCRT) among patients with incompletely resected NSCLC.

## Methods

This cohort study was performed under a protocol approved by the Roswell Park Comprehensive Cancer Center, with a waiver of informed consent because the research met the criteria for minimal risk to study participants. The study followed the Strengthening the Reporting of Observational Studies in Epidemiology (STROBE) reporting guideline.

The NCDB was queried for patients diagnosed between January 2004 and December 2019 with nonmetastatic NSCLC who underwent surgery with positive margins followed by either IMRT or 3DCRT for PORT. Variables of interest included facility type, age, race, insurance type, income,^7^ Charlson-Deyo comorbidity score, year of diagnosis, histologic features, tumor grade, T and N stage, surgery, surgical margin, radiation therapy, and chemotherapy. Race was self-reported during the initial assessment and was included to evaluate whether racial differences exist in undergoing IMRT. All missing values were coded as unknown. Clinically pertinent variables, including medical comorbidities, performance status, type and duration of systemic therapy, toxic effect profile, tumor recurrence, and lung cancer–specific mortality, were not captured in the NCDB.

### Statistical Analysis

The primary end points were OS, defined as the time between diagnosis and the last follow-up or death. Baseline characteristics between the 3DCRT and IMRT arms were compared using Fisher exact test or Mann-Whitney U test as appropriate. Cochran-Armitage test was performed to evaluate the temporal trend of IMRT from 2004 to 2019. Kaplan-Meier method, log-rank test, and multivariable Cox proportional hazards regression analysis were performed to evaluate the association of IMRT with OS compared with 3DCRT. Interaction term analysis was performed to evaluate the heterogeneous association of IMRT with OS. Survival data for patients diagnosed with NSCLC in 2019 were not captured in the NCDB, and these patients were not included for analysis of OS. Multivariable logistic regression analysis was performed to identify variables associated with IMRT. These models included the aforementioned clinically relevant variables.

To reduce selection bias, propensity score matching was performed based on all variables of interest. Matching was performed using a nearest-neighbor method in a 1:1 ratio without replacements. The standardized differences of all variables were lower than 0.1, indicating adequate match.^8^ To exclude patients who died after surgery, sensitivity analysis was performed by repeating the multivariable Cox proportional hazards regression analysis among patients with postdiagnosis survival of more than 4 months. In addition, patients included in our study were diagnosed over the span of more than a decade with different American Joint Committee on Cancer (AJCC) *Cancer Staging Manual* editions, which may have led to misclassification in staging for select patients. Multivariable Cox proportional hazards regression was also repeated among patients diagnosed based on the AJCC *Cancer Staging Manual, Seventh Edition* only.

All *P* values were 2-sided, and *P* < .05 was considered statistically significant. Analyses were performed using R, version 4.0.3 (R Project for Statistical Computing).

## Results

**Figure 1.**
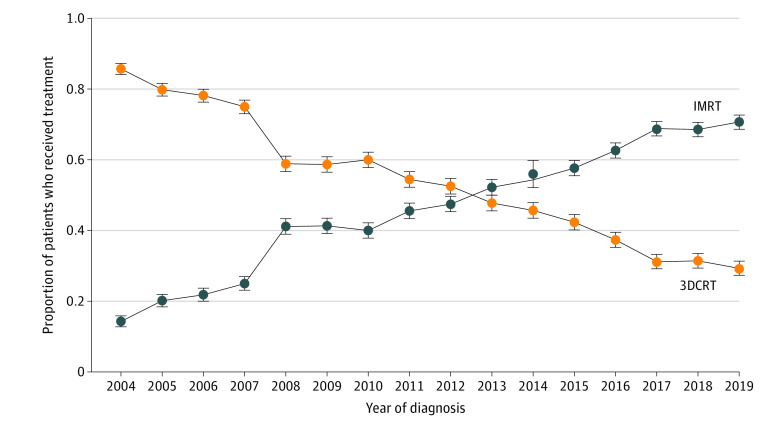
Trends in Use of Intensity-Modulated Radiation Therapy (IMRT) vs 3D Conformal Radiation Therapy (3DCRT) From January 2004 to December 2019 Error bars represent 95% CIs.

A total of 4483 patients (2439 men [54.4%]; median age, 67 years [IQR, 60-73 years]) met our inclusion criteria (Table 1). Of those, 2116 (47.2%) underwent 3DCRT and 2367 (52.8%) underwent IMRT. Median follow-up was 48.5 months (IQR, 31.1-77.2 months). The proportion of patients who underwent IMRT increased from 14.3% (13 of 91 patients) in 2004 to 70.7% (333 of 471 patients) in 2019 (*P* < .001) (Figure 1).

**Table 1.** Baseline Characteristics of Patients With Incompletely Resected Non–Small Cell Lung Cancer Who Received IMRT or 3DCRT for Postoperative Radiation Therapy

Variable	Before propensity score matching			After propensity score matching		
	Patients, No. (%)		*P* value	Patients, No. (%)		*P* value
	3DCRT (n = 2116)	IMRT (n = 2367)		3DCRT (n = 1463)	IMRT (n = 1463)	
**Patient characteristics**						
Treatment facility type						
Nonacademic	1553 (73.4)	1649 (69.7)	.01	1044 (71.4)	1052 (71.9)	.96
Academic	542 (25.6)	699 (29.5)		407 (27.8)	399 (27.3)	
Not available	21 (1.0)	19 (0.8)		12 (0.8)	12 (0.8)	
Age, y						
<65	894 (42.2)	940 (39.7)	.09	617 (42.2)	630 (43.1)	.65
≥65	1222 (57.8)	1427 (60.3)		846 (57.8)	833 (56.9)	
Sex						
Female	954 (45.1)	1090 (46.0)	.53	673 (46.0)	675 (46.1)	.97
Male	1162 (54.9)	1277 (54.0)		790 (54.0)	788 (53.9)	
Year of diagnosis, median (IQR)	2012 (2009-2016)	2015 (2012-2018)	<.001	2013 (2010-2016)	2013 (2010-2016)	.65
Race						
Black	210 (9.9)	212 (9.0)	.01	145 (9.9)	133 (9.1)	.78
White	1864 (88.1)	2071 (87.5)		1284 (87.8)	1293 (88.4)	
Other^a^	37 (1.7)	75 (3.2)		31 (2.1)	32 (2.2)	
Not available	5 (0.2)	9 (0.4)		3 (0.2)	5 (0.3)	
Insurance type						
Not insured	44 (2.1)	37 (1.6)	.34	30 (2.1)	27 (1.8)	.99
Government^b^	1422 (67.2)	1637 (69.2)		972 (66.4)	977 (66.8)	
Private	636 (30.1)	675 (28.5)		450 (30.8)	448 (30.6)	
Not available	14 (0.7)	18 (0.8)		11 (0.8)	11 (0.8)	
Income^c^						
Above median	973 (46.0)	1105 (46.7)	.01	677 (46.3)	680 (46.5)	.95
Below median	890 (42.1)	918 (38.8)		592 (40.5)	595 (40.7)	
Not available	253 (12.0)	344 (14.5)		194 (13.3)	188 (12.9)	
Charlson-Deyo comorbidity score						
0 or 1	1759 (83.1)	1962 (82.9)	.84	1217 (83.2)	1211 (82.8)	.81
>1	357 (16.9)	405 (17.1)		246 (16.8)	252 (17.2)	
**Tumor characteristics**						
Site						
Upper lobe	1253 (59.2)	1348 (56.9)	.11	851 (58.2)	852 (58.2)	.98
Middle lobe	93 (4.4)	124 (5.2)		64 (4.4)	68 (4.6)	
Lower lobe	522 (24.7)	641 (27.1)		376 (25.7)	374 (25.6)	
Other	248 (11.7)	254 (10.7)		172 (11.8)	169 (11.6)	
Side						
Right	1129 (53.4)	1244 (52.6)	.07	778 (53.2)	782 (53.5)	.99
Left	859 (40.6)	1012 (42.8)		606 (41.4)	603 (41.2)	
Other	128 (6.0)	111 (4.7)		79 (5.4)	78 (5.3)	
Grade^d^						
I	90 (4.3)	103 (4.4)	<.001	76 (5.2)	66 (4.5)	.71
II	675 (31.9)	623 (26.3)		486 (33.2)	490 (33.5)	
III	825 (39.0)	726 (30.7)		571 (39.0)	554 (37.9)	
IV	47 (2.2)	38 (1.6)		29 (2.0)	36 (2.5)	
Not available	479 (22.6)	877 (37.1)		301 (20.6)	317 (21.7)	
Histologic features						
Squamous	881 (41.6)	903 (38.1)	.02	589 (40.3)	600 (41.0)	.71
Nonsquamous	1235 (58.4)	1464 (61.9)		874 (59.7)	863 (59.0)	
T stage						
1	267 (12.6)	246 (10.4)	<.001	200 (13.7)	186 (12.7)	.94
2	512 (24.2)	474 (20.0)		369 (25.2)	369 (25.2)	
3	496 (23.4)	499 (21.1)		370 (25.3)	383 (26.2)	
4	191 (9.0)	238 (10.1)		153 (10.5)	156 (10.7)	
Not available	650 (30.7)	910 (38.4)		371 (25.4)	369 (25.2)	
N stage						
0 or 1	1036 (49.0)	946 (40.0)	<.001	747 (51.1)	761 (52.0)	.83
2 or 3	325 (15.4)	398 (16.8)		263 (18.0)	252 (17.2)	
Not available	755 (35.7)	1023 (43.2)		453 (31.0)	450 (30.8)	
**Treatment**						
Chemotherapy						
None	512 (24.2)	476 (20.1)	.003	322 (22.0)	327 (22.4)	.94
Single agent	90 (4.3)	86 (3.6)		59 (4.0)	54 (3.7)	
Multiple agents	1298 (61.3)	1573 (66.5)		937 (64.0)	931 (63.6)	
Other	216 (10.2)	232 (9.8)		145 (9.9)	151 (10.3)	
Surgery						
Sublobar resection	604 (28.5)	646 (27.3)	.28	414 (28.3)	423 (28.9)	.93
Lobectomy	1190 (56.2)	1361 (57.5)		829 (56.7)	812 (55.5)	
Pneumonectomy	139 (6.6)	179 (7.6)		103 (7.0)	105 (7.2)	
Other	183 (8.6)	181 (7.6)		117 (8.0)	123 (8.4)	
Surgical margin						
Other	771 (36.4)	951 (40.2)	.02	547 (37.4)	549 (37.5)	.99
Microscopic	1110 (52.5)	1194 (50.4)		763 (52.2)	763 (52.2)	
Macroscopic	235 (11.1)	222 (9.4)		153 (10.5)	151 (10.3)	

Abbreviations: 3DCRT, 3D conformal radiation therapy; IMRT, intensity modulated radiation therapy.

^a^ “Other” includes, but is not limited to, American Indian, Asian, and Pacific Islander. They were grouped together because the sample size of each subgroup was too small for analysis.

^b^ Includes Medicaid and Medicare.

^c^ Based on the median household income adjusted for 2016 inflation ($50 353) in each patient’s zip code, according to 2016 American Community Survey data.^7^

^d^ Grade I indicates well differentiated tumor; II, moderately differentiated; III, poorly differentiated; and IV, undifferentiated.

On multivariable Cox proportional hazards regression, IMRT was associated with improved OS compared with 3DCRT (5-year OS, 38.2% vs 31.9%; adjusted hazard ratio [aHR], 0.84; 95% CI, 0.78-0.91; *P* < .001). Having nonsquamous tumors (aHR, 0.87; 95% CI, 0.81-0.95; *P* = .001) and receipt of a recent diagnosis (aHR, 0.98; 95% CI, 0.97-0.99; *P* < .001), lobectomy (aHR, 0.87; 95% CI, 0.79-0.96; *P* = .007), multi-agent chemotherapy (aHR, 0.81; 95% CI, 0.73-0.89; *P* < .001), and treatment at an academic facility (aHR, 0.81; 95% CI, 0.74-0.88; *P* < .001) were associated with improved OS (Table 2). Age 65 years or older, male sex, worse comorbidities, and presence of grade 3 tumors, T3 and T4 stage tumors, and N2 and N3 stage tumors were associated with worse OS (Table 2). There was no statistically significant interaction with T stage (interaction *P* = .86) or N stage (interaction *P* = .57), surgical margin status (interaction *P* = .35), or histologic features (interaction *P* = .93).

**Table 2.** Multivariable Cox Proportional Hazards Regression and Logistic Regression Analyses for the Association of Variables With Overall Survival and Intensity-Modulated Radiation Therapy

Variable	Cox proportional hazards regression		Logistic regression	
	aHR (95% CI)	*P* value	aOR (95% CI)	*P* value
**Patient characteristics**				
Treatment facility type				
Nonacademic	1.00 [Reference]	NA	1.00 [Reference]	NA
Academic	0.81 (0.74-0.88)	<.001	1.15 (1.00-1.33)	.049
Age, y				
<65	1.00 [Reference]	NA	1.00 [Reference]	NA
≥65	1.26 (1.14-1.39)	<.001	1.12 (0.96-1.31)	.16
Sex				
Female	1.00 [Reference]	NA	1.00 [Reference]	NA
Male	1.18 (1.09-1.28)	<.001	0.95 (0.84-1.09)	.48
Year of diagnosis				
For every 1-y increase	0.98 (0.97-0.99)	<.001	1.17 (1.15-1.19)	<.001
Race				
Black	1.05 (0.91-1.20)	.50	0.86 (0.69-1.07)	.19
White	1.00 [Reference]	NA	1.00 [Reference]	NA
Other^a^	0.72 (0.54-0.97)	.03	1.53 (1.00-2.36)	.05
Insurance type				
Not insured	1.00 [Reference]	NA	1.00 [Reference]	NA
Government^b^	1.17 (0.85-1.61)	.32	1.26 (0.78-2.04)	.35
Private	1.05 (0.77-1.44)	.77	1.2 (0.75-1.94)	.45
Income^c^				
Above median	1.00 [Reference]	NA	1.00 [Reference]	NA
Below median	0.98 (0.90-1.06)	.57	0.95 (0.83-1.09)	.45
Charlson-Deyo comorbidity score				
0 or 1	1.00 [Reference]	NA	1.00 [Reference]	NA
>1	1.17 (1.06-1.29)	.002	1.01 (0.85-1.20)	.90
**Tumor characteristics**				
Site				
Upper lobe	1.00 [Reference]	NA	1.00 [Reference]	NA
Middle lobe	1.00 (0.82-1.21)	.99	1.32 (0.97-1.80)	.08
Lower lobe	1.15 (1.05-1.26)	.002	1.10 (0.95-1.28)	.21
Other	1.13 (0.96-1.32)	.14	1.05 (0.80-1.37)	.72
Side				
Right	1.00 [Reference]	NA	1.00 [Reference]	NA
Left	1.00 (0.92-1.09)	.96	1.08 (0.95-1.24)	.24
Other	0.95 (0.75-1.19)	.64	0.71 (0.49-1.04)	.08
Grade^d^				
I	1.00 [Reference]	NA	1.00 [Reference]	NA
II	1.11 (0.92-1.34)	.28	0.74 (0.53-1.02)	.07
III	1.32 (1.09-1.59)	.004	0.75 (0.54-1.04)	.08
Other	1.37 (1.01-1.87)	.04	0.61 (0.35-1.06)	.08
Histologic features				
Squamous	1.00 [Reference]	NA	1.00 [Reference]	NA
Nonsquamous	0.87 (0.81-0.95)	.001	1.15 (1.01-1.32)	.04
T stage				
1	1.00 [Reference]	NA	1.00 [Reference]	NA
2	1.14 (1.00-1.30)	.06	0.99 (0.78-1.24)	.91
3	1.37 (1.19-1.57)	<.001	1.07 (0.85-1.35)	.56
4	1.31 (1.11-1.54)	.001	1.50 (1.13-1.99)	.005
N stage				
0 or 1	1.00 [Reference]	NA	1.00 [Reference]	NA
2 or 3	1.44 (1.30-1.60)	<.001	1.25 (1.04-1.51)	.02
**Treatment**				
Chemotherapy				
None	1.00 [Reference]	NA	1.00 [Reference]	NA
Single agent	1.16 (0.95-1.41)	.15	1.13 (0.79-1.60)	.50
Multiple agents	0.81 (0.73-0.89)	<.001	1.20 (1.02-1.42)	.03
Other	1.09 (0.95-1.25)	.23	1.32 (1.04-1.69)	.02
Surgery				
Sublobar resection	1.00 [Reference]	NA	1.00 [Reference]	NA
Lobectomy	0.87 (0.79-0.96)	.007	1.02 (0.87-1.20)	.78
Pneumonectomy	0.97 (0.81-1.15)	.70	1.35 (1.02-1.80)	.04
Other	1.58 (1.34-1.87)	<.001	0.85 (0.65-1.12)	.26
Surgical margin				
Other	1.00 [Reference]	NA	1.00 [Reference]	NA
Microscopic	0.85 (0.78-0.92)	<.001	0.88 (0.77-1.01)	.08
Macroscopic	1.04 (0.91-1.19)	.56	0.91 (0.73-1.15)	.44

Abbreviations: aHR, adjusted hazard ratio; aOR, adjusted odds ratio.

^a^ “Other” includes, but is not limited to, American Indian, Asian, and Pacific Islander. They were grouped together because the sample size of each subgroup was too small for analysis.

^b^ Includes Medicaid and Medicare.

^c^ Based on the median household income adjusted for 2016 inflation ($50 353) in each patient’s zip code, according to 2016 American Community Survey data.^7^

^d^ Grade I indicates well differentiated tumor; II, moderately differentiated; III, poorly differentiated; and IV, undifferentiated.

**Figure 2.**
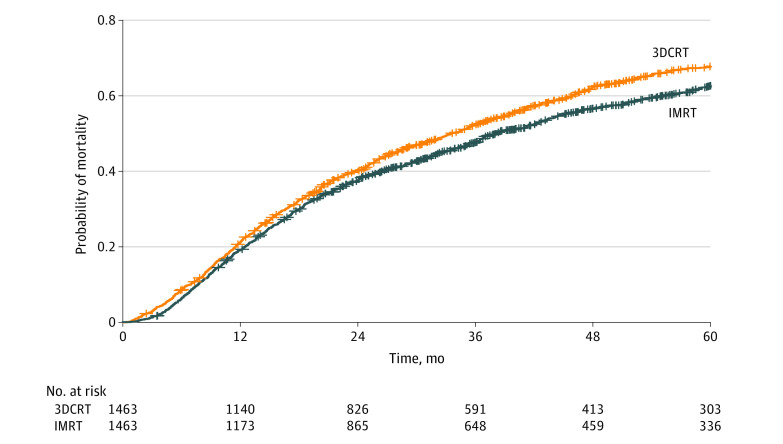
Cumulative Hazard of Overall Mortality Between Patients Who Received Intensity-Modulated Radiation Therapy (IMRT) vs 3D Conformal Radiation Therapy (3DCRT) After Propensity Score Matching

After propensity score matching, 1463 matched pairs were identified. All variables were well balanced (Table 1). Compared with 3DCRT, IMRT was associated with improved 5-year OS (37.3% vs 32.2%; HR, 0.88; 95% CI, 0.80-0.96; *P* = .003) (Figure 2).

On multivariable logistic regression analysis (Table 2), patients were more likely to receive IMRT if they had received a recent diagnosis of NSCLC (adjusted odds ratio [aOR], 1.17; 95% CI, 1.15-1.19; *P* < .001), had nonsquamous tumors (aOR, 1.15; 95% CI, 1.01-1.32; *P* = .04), received treatment at an academic facility (aOR, 1.15; 95% CI, 1.00-1.33; *P* = .049), underwent pneumonectomy (aOR, 1.35; 95% CI, 1.02-1.80; *P* = .04), received multi-agent chemotherapy (aOR, 1.20; 95% CI, 1.02-1.42; *P* = .03), and had N2 or N3 stage tumors (aOR, 1.25; 95% CI, 1.04-1.51; *P* = .02) and T4 stage tumors (aOR, 1.50; 95% CI, 1.13-1.99; *P* = .005). On sensitivity analysis, similar survival outcomes for IMRT were observed among 3883 patients (86.6%) with postdiagnosis survival of more than 4 months (aHR, 0.87; 95% CI, 0.80-0.95; *P* = .001) and among 2575 patients (57.4%) with staging based on the AJCC *Cancer Staging Manual, Seventh Edition* only (aHR, 0.82; 95% CI, 0.74-0.90; *P* < .001).

## Discussion

To our knowledge, this is the first study using a nationwide clinical oncology database to report an OS benefit associated with IMRT compared with 3DCRT for PORT among patients with incompletely resected NSCLC. The survival benefit associated with IMRT in our study is consistent with prior literature of locally advanced NSCLC, suggesting that the survival benefit^9^ may in part be associated with lower pulmonary toxic effects and cardiac doses.^10^ Our finding of improved OS associated with IMRT is also consistent with results of the recently reported LungART and PORT-C trials, although both excluded patients with positive margins.^4,5^ The PORT-C trial used IMRT in 90% of patients and reported improved disease-free survival among per-protocol patients without any grade 4 or 5 toxic effects.^4^ The LungART trial, however, used IMRT in only 10% of patients and reported higher rates of cardiopulmonary toxic effects after PORT.^5^

Because the bronchial stump has been shown to be the most common treatment failure site, with high mediastinal failure rates regardless of initial N stage,^11^ the National Comprehensive Cancer Network guideline recommends that the clinical target volume covers the postsurgical areas and high-risk draining lymph node stations.^3^ The extent and proximity of clinical target volume to organs at risk may also explain our findings that patients with higher tumor stage and those who underwent pneumonectomy were more likely to undergo IMRT as well as the increasing trend in IMRT use over time. Consistent with a prior study,^6^ the Cox proportional hazards regression analysis in our study found that IMRT was associated with improved OS regardless of stage of incompletely resected NSCLC.

Beyond IMRT, new methods of limiting the toxic effects of PORT continue to be explored. Proton therapy for PORT is associated with comparable oncologic outcomes and improved toxic effect profiles compared with IMRT.^12,13,14^ A prospective trial investigating postoperative stereotactic body radiation therapy is currently ongoing.^15^

### Limitations

This study has limitations, including the use of retrospective data. Although extracapsular extension was shown to be associated with worse survival,^16^ such high-risk features were not captured in the NCDB. In addition, performance status, cardiopulmonary toxic effect profiles, and tumor recurrence outcomes were also unavailable in the NCDB, and selection bias may have occurred despite multivariable analyses and propensity score matching. Furthermore, because all patients included in the cohort received a diagnosis of NSCLC before the publication of the CheckMate 816 trial,^2^ many of them did not receive immunotherapy. Our findings may need to be validated further based on a more recent patient cohort.

## Conclusions

In this cohort study, use of IMRT for PORT among patients with incompletely resected NSCLC increased in the US from 2004 to 2019 and was associated with improved survival outcomes compared with 3DCRT. Further studies are warranted to investigate the role of IMRT for PORT.
